# Initial Experience of Endoscopic Phonosurgery With a Prototype of the Therapeutic Rhinolarynx Electronic Endoscope

**DOI:** 10.1155/DTE.1.229

**Published:** 1995

**Authors:** Masahiro Kawaida, Hiroyuki Fukuda, Akihiro Shiotani, Naoyuki Kohno

**Affiliations:** 1 Department of Otolaryngology Tokyo Metropolitan Ohtsuka Hospital Tokyo, Japan 8-1, 2-chome Minamiohtsuka Toshima-ku Tokyo 170 Japan; 2 Department of Otolaryngology Keio University School of Medicine Tokyo, Japan 35 Shinanomachi Shinjuku-ku Tokyo 160 Japan; 3 Department of Otolaryngology Juntendo University School of Medicine Tokyo, Japan 1-1, 2-chome Hongo Bunkyo-ku Tokyo 113 Japan

## Abstract

We performed endoscopic phonosurgery in a patient with a laryngeal lesion using a prototype of the therapeutic rhino-larynx electronic endoscope connected to a video processor (Asahi Optical Co., Ltd.). This therapeutic electronic endoscope differs from the fiberoptic endoscope, because it contains an instrument channel and a miniature television camera attached to the tip of the endoscope, consisting of a small light-sensitive CCD chip. The dynamic image provided by this system is superior in resolution to that obtained by conventional flexible laryngofiberscopes. Using this therapeutic electronic endoscope and flexible forceps, we succeeded in removing a vocal fold polyp. This endoscope can be passed through the nasal passage into the laryngeal cavity. The therapeutic electronic endoscope is introduced and a clinical case is presented.


*Diagnostic and Therapeutic Endoscopy*, 1995, Vol. 1, pp. 229–232
Reprints available directly from the publisher
Photocopying permitted by license only

© 1995 Harwood Academic Publishers GmbH
Printed in Singapore

### Initial Experience of Endoscopic Phonosurgery with a Prototype of the Therapeutic Rhinolarynx Electronic Endoscope

MASAHIRO KAWAIDA,* HIROYUKI FUKUDA,† AKIHIRO SHIOTANI,† and NAOYUKI KOHNO‡

**Department of Otolaryngology, Tokyo Metropolitan Ohtsuka Hospital, Tokyo, Japan 8-1, 2-chome Minamiohtsuka, Toshima-ku, Tokyo 170, Japan; †Department of Otolaryngology, Keio University School of Medicine, Tokyo, Japan 35 Shinanomachi, Shinjuku-ku, Tokyo 160, Japan; ‡Department of Otolaryngology, Juntendo University School of Medicine, Tokyo, Japan 1-1, 2-chome Hongo, Bunkyo-ku, Tokyo 113, Japan*

*(Received June 22, 1994; in final form September 25, 1994)*

We performed endoscopic phonosurgery in a patient with a laryngeal lesion using a prototype of the therapeutic rhino-larynx electronic endoscope connected to a video processor (Asahi Optical Co., Ltd.). This therapeutic electronic endoscope differs from the fiberoptic endoscope, because it contains an instrument channel and a miniature television camera attached to the tip of the endoscope, consisting of a small light-sensitive CCD chip. The dynamic image provided by this system is superior in resolution to that obtained by conventional flexible laryngofiberscopes. Using this therapeutic electronic endoscope and flexible forceps, we succeeded in removing a vocal fold polyp. This endoscope can be passed through the nasal passage into the laryngeal cavity. The therapeutic electronic endoscope is introduced and a clinical case is presented.

**KEY WORDS:** therapeutic rhino-larynx electronic endoscope, therapeutic laryngoendoscope, phonosurgery, vocal fold polyp, RGB sequencing system

### INTRODUCTION

An electronic endoscope has been developed with a small CCD (charge coupled device) camera attached to the tip, allowing for the precise visualization of laryngeal lesions and determination of appropriate treatment. We used a prototype of the therapeutic rhinolarynx electronic endoscope for the phonosurgical resection of vocal fold polyps. We describe the techniques and procedures of endoscopic phonosurgery with a prototype of this endoscope system to obtain a smooth insertion through the nasal passage into the laryngeal cavity.

### INSTRUMENTS AND METHODS

Endoscopic phonosurgery was performed using a prototype of the therapeutic rhinolarynx electronic endoscope PENTAX VNL-2000 (Asahi Optical Co., Ltd.) (Fig. 1). The dimensions and characteristics of this endoscope are shown in Table 1. It contains a small light-sensitive CCD chip, acting as a miniature television camera that is attached to the tip of the endoscope. The endoscope transforms the dynamic images detected by the CCD chip into electronic signals, and a video processor PENTAX EPM-3300 (Asahi Optical Co., Ltd.) changes these signals into images that can be visualized on a television monitor by the RGB sequencing system. A flexible forceps can be inserted through the instrument channel, which measures 2 mm in inner diameter (Fig. 1). Light source for illumination is included in the video processor. Other features, such as the control mechanisms of the tip, resemble those of conventional therapeutic flexible laryngofiberscopes.

This electronic endoscope is used on patients in the seated position after administering surface anesthesia to the nasal and laryngeal cavities using cotton applicators soaked in 4% lidocaine-HCl. The endoscope then is passed through the nasal passage and introduced into the laryngeal cavity.

Address for correspondence: Dr. Masahiro Kawaida, Department of Otolaryngology, Tokyo Metropolitan Ohtsuka Hospital, 8-1, 2-chome Minamiohtsuka, Toshima-ku, Tokyo 170, Japan

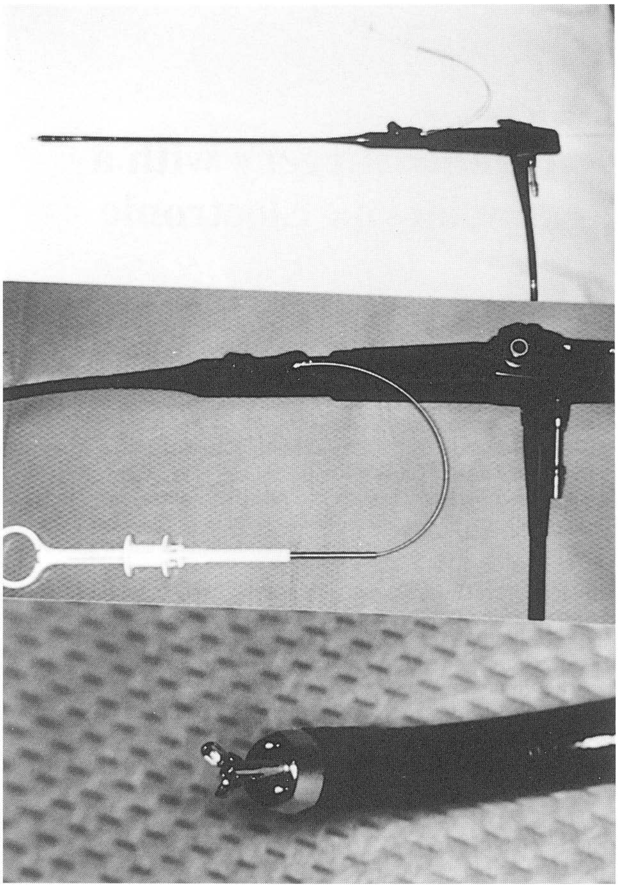

**Figure 1** A prototype of the therapeutic rhinolarynx electronic endoscope and flexible forceps as a endoscopic phonosurgical product (Asahi Optical Co., Ltd.)

### PATIENT

A 79-year-old man had been suffering from hoarseness for 5 weeks before visiting our clinic on May 6, 1993. A right vocal fold polyp was diagnosed with a conventional flexible laryngofiberscope (Fig. 2). Because of his advanced age and presence of cardiac insufficiency, we conducted endoscopic phonosurgery using the therapeutic rhinolarynx electronic endoscope system.

**Table 1** A prototype of the therapeutic rhinolarynx electronic endoscope (Asahi Optical Co., Ltd.) dimensions and characteristics

| | | |
|---|---|---|
| *Optical system* | *Field of View*<br>*Direction of View*<br>*Depth of Field* | *120°*<br>*Forward viewing*<br>*5–100 mm* |
| Bending section | Range of tip bending | Up 180°, down 130° |
| Distal end | Maximum length in outer diameter of oval shape | 7.5 mm |
| Insertion tube | Outer diameter | 6.5 mm |
| Instrument channel | Inner diameter | 2.0 mm |
| Working length | | 300 mm |
| Total length | | 515 mm |
| Video processer | | PENTAX EPM-3300 (RGB seq. system) |

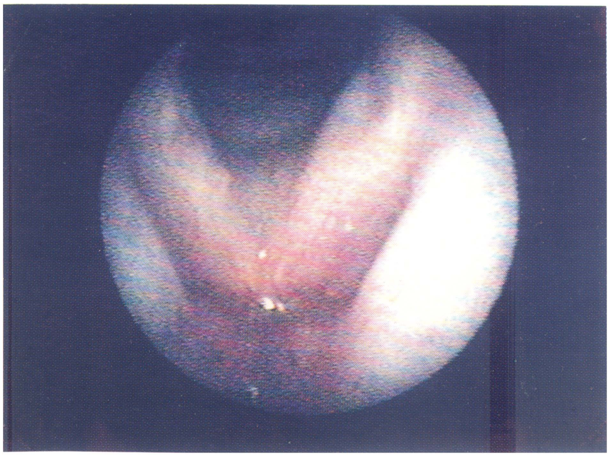

**Figure 2** Preoperative findings of the patient with the right vocal fold polyp observed by using a conventional flexible laryngofiberscope.

### RESULTS

After introducing the endoscope into the laryngeal cavity through the nasal passage, we observed a polypous lesion on the midportion of the right vocal fold (Fig. 3). A flexible forceps was inserted into the instrument channel of the scope (Fig. 4A). The lesion was grasped and excised (Fig. 4B and C). Postoperative findings showed complete resection of the lesion (Fig. 4D).

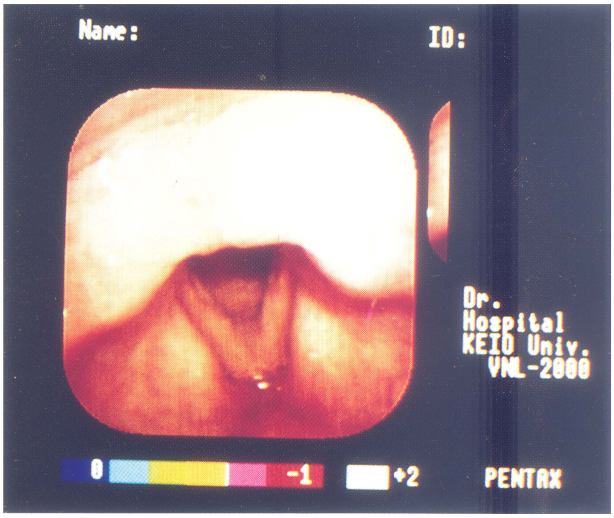

**Figure 3** Preoperative findings of the patient with the right vocal fold polyp observed by using a this electronic endoscope.

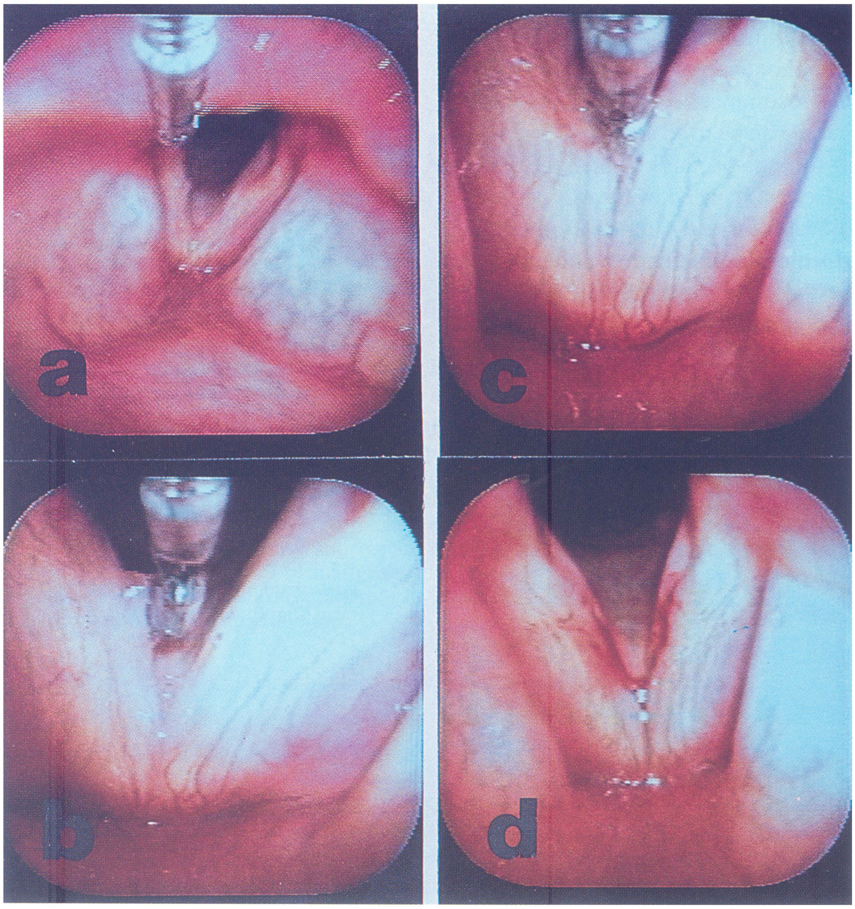

**Figure 4** Endoscopic phonosurgical findings in this patient with a prototype of the therapeutic rhino-larynx electronic endoscope
a, A flexible forceps was pushed through the instrument channel of the scope.
b, The polypous lesion was grasped by a forceps.
c, The polypous lesion grasped by a forceps was drawn together.
d, The vocal fold polyp was resected precisely.

The quality and resolution of the dynamic images obtained with this electronic endoscope system were superior to those obtained with a conventional flexible laryngofiberscope. The maneuverability of this electronic endoscope is easy and identical to that of a conventional flexible laryngofiberscope.

### DISCUSSION

Laryngomicrosurgery performed under a direct laryngoscope has been successful in the phonosurgical treatment of laryngeal lesions (1–3). A therapeutic flexible laryngofiberscope has also been used to perform minor surgery on such laryngeal lesions as vocal fold polyps (4).

We performed endoscopic phonosurgery on a vocal fold polyp using a prototype of the therapeutic rhinolarynx electronic endoscope. The mechanical characteristics of this electronic endoscope are almost identical to those of flexible laryngofiberscopes, but the electronic endoscope with monitoring system contains a small light-sensitive CCD chip at its tip (5–8). This endoscope system provides higher quality, optimum brightness, superior color balance, and high resolution of the television image.

This prototype of the therapeutic rhinolarynx electronic endoscope contained an instrument channel and a small oval-shaped CCD camera at its tip with a maximum outer diameter of 7.5 mm. This electronic endoscope can be introduced through the nasal passage into the laryngeal cavity after sufficient surface anesthesia has been applied. Patients experience less pain with this instrument, and the dynamic images provided by this system are superior to those obtained by conventional flexible laryngofiberscopes in terms of resolution and detail. The main advantage is its high-quality video image reproduction on a television monitor. Better definition of image aids the performance of endoscopic phonosurgery.

This device is highly recommended for the following additional reasons. First, it can be used even with patients who cannot open their mouths because of odontological problems. Second, it also can be used even with patients who have severe cardiovascular and pulmonary disease. Finally, it can be performed in ambulatory patients administered surface anesthesia to the nasal and laryngeal cavities without hospital admission.

### CONCLUSION

A prototype of the therapeutic rhinolarynx electronic endoscope was introduced through the nasal passage into the laryngeal cavity to perform endoscopic phonosurgery under surface anesthesia. Results demonstrated that this electronic endoscope system provided high quality and high resolution of the television images.

### REFERENCES

1. Saito S, Fukuda H, Kitahara S. Stroboscopic microsurgery of the larynx. Arch Otolaryngol 1975;101:196–201
2. Saito S. Phonosurgery: basic study on the mechanism of phonation and endolaryngeal microsurgery. Otologia Fukuoka 1977;23:171–384
3. Kawaida M, Fukuda H, Kano S, et al.: Laryngomicrosurgery by use of a side-opened direct laryngoscope. In: Inouye T, Fukuda H, Sato T, Hinohara T, (eds.): *Recent Advances in Bronchoesophagology*, Elsevier Science Publishers B.V., Amsterdam, 1990;355–356
4. Fukuda H, Kawaida M, Kano S, et al. Stroboscopic phonosurgical operation by using a therapeutic flexible laryngofiberscope. J Jpn Bronchoesophagol Soc 1989;40:240–245
5. Classen M, Phillip J. Electronic endoscopy of the gastrointestinal tract: initial experience with a new type of endoscope that has no fiberoptic bundle for imaging. Endoscopy 1984;16:16–19
6. Matek W, Lux G, Riemann JF, et al. Initial experience with the electronic endoscope. Endoscopy 1984;16:20–21
7. Niwa H, Kawaguchi A, Miyahara T, et al. Clinical use of new videoendoscopes (EVIS 100 and 200). Endoscopy 1992;24:222–224
8. Kawaida M, Fukuda H, Kohno N. Clinical experience with a new type of rhino-larynx electronic endoscope PENTAX VNL-1530. Diag Ther Endoscopy 1944;1:57–62

